# A Jungle Hospital

**Published:** 1917-09-01

**Authors:** 


### A JUNGLE HOSPITAL.

### II. Causes of Plague, Former Residencies, and Some Sport.

As is well known, plague reappears among the native population of India during every cold weather, thousands dying of the disease every year. The infection is transmitted to human beings from plague-infected rats, by means of the bite of the common rat flea. During the cold weather the people huddle together in miserable mud-houses and infection spreads with alarming rapidity. Driving through the district it is easy to tell the villages where plague is really bad. The houses are empty, and along the roads near are huts of grass or wood built by the fugitives who are afraid to remain in the infected villages. Wherever plague is known to exist the Plague Sahib goes, making inquiries, and urging the people to leave their houses for huts, and to submit to inoculation against the disease. In some villages it is easy to persuade the people to take these efficient preventive measures. In others they are too conservative to welcome any fresh idea. They hesitate to leave their houses lest their possessions are stolen in their absence, or they are afraid that inoculation will bring other diseases in its train, and to all persuasion and exhortation will only give the same answer, "Plague comes every year. It is the will of Allah."

Here and there a rajah, or an influential native land-owner, more enlightened than his neighbours, will invite the Plague Sahib to come to his town or village and speak to the people, sending his car to convey him, and offering such hospitality as he thinks will be welcome. He is often the first to be inoculated, when others, ashamed to refuse, or dimly conscious of some benefit to follow, will submit to inoculation. In this way many hundreds of people have been reached, and saved from the fatal disease. Inoculation is never compulsory but the natives are so like children in many ways, that with a little talk and banter, and the exercise of much tact, quite a number can often be persuaded to take this precautionary measure. Wherever possible, plague patients are treated in their own houses, or medicines and other relief sent to them.

As soon as the work undertaken in one part of the district is completed, camp is moved to the next spot chosen, and during the winter's camping probably five or six long halts are made, varying in length from one to three weeks. The other halts are often for not more than one night or two, and sometimes five or six consecutive marches have to be undertaken. The marches are done by motor or motor-cycle, except when the march is across country, when elephants or "ekkas" are used. An ekka is a native-made conveyance drawn by a more or less decrepit pony. The shafts are of bamboo, and above the wheels is a board on which the passenger sits sideways. At first they seem most uncomfortable, but they are a feature of every Indian district, and one gets quite used to them. The ponies often have bells attached to them, and their pleasant jingle is heard on every road in India.

When the day's work is over, the Plague Sahib and his family must find their evening's recreation round about the camp, unless they are near enough to a station to be able to motor in for a game of tennis. As this only happens occasionally we generally go for a walk, and it is astonishing how interesting such an apparently dull prospect may become. Sometimes we cross the fields, roughly sown with sugar-cane or barley, to a village, to make inquiries about plague or the chances of inoculation; sometimes we only wander up and down the grove under the wide-spreading branches of the mango-trees, or stroll to the nearest pond to give the dogs a swim. Occasionally we find an old forsaken bungalow where one may still see the indigo-vats overgrown with weeds and shrubs, or, looking through the windows, see chairs and tables and children's toys as they were left years ago. There is always some old man in charge who remembers about his old master, and who will tell us that "the Sahib died many years ago, and the mem-sahib went to England with the baba log." We always wondered where they were, and if the mem-sahib, grown old in England, ever remembered the wide verandahs and rose-bordered paths of her Indian home. If we were near a large town or village we always loved to drive or ride through the busy bazaars at sundown, and see the merchants sitting in shops lit by one small and smoky lamp. Those evening smells of hot fat and burning wood that Kipling says are in every corner of the world as day dies, came out of the houses and filled the narrow streets, crowded with softly moving natives. Here and there a bright flare showed where a sweet-maker was busy making piles of yellow "jalabies." At every door stood brightly polished brass lotas, and over it all was thrown the faint blue mist of a cold-weather evening.

If there were a snipe jheel near at hand, an hour spent there with a gun often resulted in a good bag, or in a brace of wild-duck, and a good jheel in the district will now and again afford the Sahib a whole day of sport and much-needed relaxation; while at Christmas a general reunion of district officials provides, in addition to a whole-day shoot on the best jheel in the district, a cheerful exchange of dinners, with much bridge and conversation. One never tires of riding to a shoot on an elephant. Though one is horribly jolted and swung, and the process of scrambling up with the aid of the elephant's foot and its tail held round to form a step is rather trying at first, one sees the country from an absolutely new point of view. The huge beasts, bearing aloft the mem-sahibs in topees and veils, swing out of the camping-ground, and down the dusty roads, through narrow, narrow village streets where we look down on the mud roofs of the houses

☛ The previous article appeared on August 25, p. 425.

and their doorways crowded with dark and wondering faces; through blue fields of flowering linseed that always remind us of English "skies uprising through the earth," past the little squares of sugar-cane, bits of which our steed always stops to break off and eat in passing, till we come to the wide expanse of reed-grown water where the men are to shoot. They go out on the jheel in long boats called "dug-outs," and sit on heaps of straw in the bottom of the boat. The boats are pushed along by "mallahs," or native boatmen, and the ducks shot as they fly overhead. These jheels are very lovely just at sunset, when the water reflects the gold and crimson of the sky, and the swish-swish of the boats being driven through the reeds mingles with the sound of voices borne across from surrounding villages.

The huge elephants, grey against the crimson afterglow, stand waiting by the edge of the jheel to carry us home again, and as we swing along while the silent Indian night is falling, the smoke from countless village houses hangs above the fields in low grey banks. In the distance gleam our own camp-fires, giving promise of our lamp-lit sitting-tent with its books and papers, and the company of the little campers of the next generation who are eagerly awaiting our return.

